# Supplementary figures and images for: Potential oxygen consumption and community composition of sediment bacteria in a seasonally hypoxic enclosed bay
Source: PeerJ. 2021 Aug 10;9:e11836. doi: 10.7717/peerj.11836 (PMC8362671; doi:10.7717/peerj.11836)

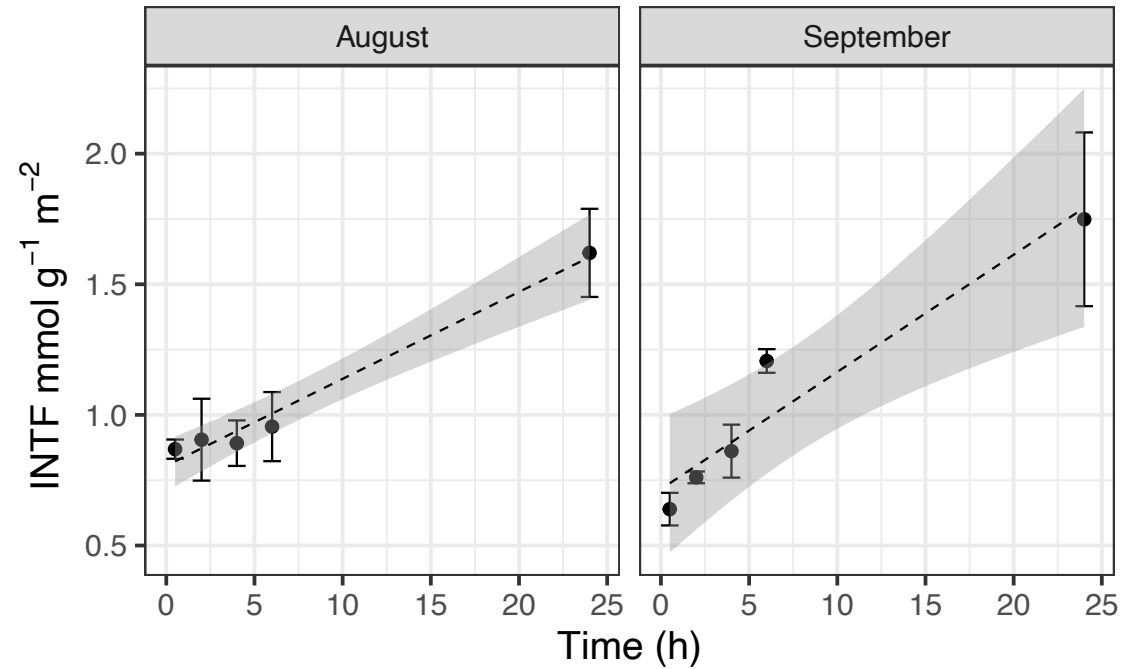

Supplement: Supplemental Information 1 — Regression line for August samples: INTF = 0.03 h + 0.81, r = 0.99, (P < 0.005). Regression line for September: INTF = 0.05 h + 0.72, r = 0.96, (P < 0.01). Error bars represent the standard error. [file peerj-09-11836-s001.pdf]
